# Rising Problems with the Term “ET-plus”: Time for the Term Makers to Go Back to the Drawing Board

**DOI:** 10.5334/tohm.555

**Published:** 2020-08-13

**Authors:** Elan D. Louis

**Affiliations:** 1 Department of Neurology and Therapeutics, University of Texas Southwestern, Dallas, TX, US

**Keywords:** essential tremor, ET-plus, clinical, diagnosis, classification

In this issue of the journal, Pandey and colleagues [1] raise concerns about the recently proposed term “ET-plus” and its implementation in various settings. As they point out, “ET-plus” includes ET patients with “questionable dystonic posturing”, a phraseology that is imprecise and difficult to operationalize in clinical and research settings. With their piece, Pandey and colleagues [1] add their voices to a growing literature that is critical of the proposed terminology [2,3,4,5,6].

Pandey and colleagues [1] detail a number of the thorny issues presented by the new classification and terminology and many of their points are worthy of consideration. Removing oneself from the weeds for a moment, however, there are several higher level issues that deserve additional comment. First, at the heart of the newly proposed classification of tremor [7] is that there is a growing awareness that ET is a disease or family of diseases that is not merely characterized by a single isolated symptom; ET is a more complex entity (or entities) and one that encompasses considerable clinical heterogeneity [8]. The recent consensus classification [7] proposed a new term and this represents an initial attempt to deal with this heterogeneity. However, it is clear that this term is a mere placeholder, that is, a temporary label [4], and the term itself and the classification it attempts to support, both require additional thought. The comments of Pandey [1] and others [2,3,4,5,6] underscore the fact that this term, “ET-plus”, was not (1) carefully defined clinically, (2) well-thought through from a pathophysiological vantage point, or (3) firmly grounded in any identifiable pharmacological or biological differences between the groups it proposed to separate. Thus, the term lacks validity [3]. Attempts to introduce defensible stratifications that are biologically-based should be careful, data-driven efforts, grounded in critical review and consideration of published data. Introducing a term as part of a consensus classification, based on the opinions of a small group of experts, is not a substitute for such an exercise.

Second, there is another high level issue. This is the reluctance of some ET scholars to recognize that the presence of dystonic movements or postures on examination does not negate an ET diagnosis. Just as a patient with Parkinson’s disease or a patient with Huntington’s disease or a patient with any number of spinocerebellar ataxias may evidence clear dystonia on examination, it is only logical that the same is true of ET, especially when one considers that patients with ET develop a plethora of motor and non-motor features as their disease advances [3,9], and that both ET and dystonia are linked to cerebellar system dysfunction [10,11]. As noted above, numerous forms of spinocerebellar ataxia are associated with dystonic features on examination [12]. This inherent bias, that the presence of dystonic movements in ET is not compatible with an ET diagnosis is what has forced the awkward terminology “questionable dystonic” posturing.

There is a third and final issue. The term “ET-plus”, rather than recognizing the heterogeneity that is present in ET, as it purports to do, is a thinly-veiled attempt to remove that very heterogeneity from ET, to cordon those patients off, and place them in another category. Ironically, that category is still given the name “ET”, so that the attempted removal of these patients from ET is not complete, leading to further vagaries. Patients with Parkinson’s disease, Huntington’s disease, and even the dystonias themselves, exhibit a panoply of motor and non-motor features, and even within the dystonias, the presence of tremor (i.e., a movement that is not sustained or twisting) is now recognized [13]. The notion that ET is a single-featured entity with only a single type of involuntary movement is no longer tenable.

In summary, there is a rising tide of voices pointing out problems with the term “ET-plus” [1,2,3,4,5,6]. It would seem that it is time for the term makers to go back to the drawing board.
